# Routine Laboratory-Based Machine Learning for Discriminating Multiple Myeloma from Clinical Mimickers: Development and Internal Validation of a Diagnostic Prediction Model

**DOI:** 10.3390/diagnostics16182928

**Published:** 2026-09-10

**Authors:** Batuhan Erdoğdu, Duygu Gül, Berrak Itır Aylı, Mine Karadeniz, Murat Yıldırım, Melda Cömert

**Affiliations:** 1 Department of Hematology, Gülhane Training and Research Hospital, Ankara 06010, Türkiye; 2 Department of Health Sciences, Hacettepe University, Ankara 06100, Türkiye

**Keywords:** multiple myeloma, machine learning, diagnostic prediction model, routine laboratory tests, clinical decision support, risk stratification, TRIPOD+AI

## Abstract

**Background:** Multiple myeloma (MM) diagnosis requires specialised testing that is not universally available, contributing to diagnostic delays. The aim of this study was to develop and internally validate statistical and machine learning (ML) models that discriminate MM from clinically similar conditions using exclusively routine laboratory parameters. **Methods:** This retrospective diagnostic prediction model study included 422 consecutive patients referred to hematology for suspected MM at a tertiary care center (2017–2025). Fifteen routine demographic and laboratory variables were used to train seven ML algorithms and two ensemble methods. Model performance was evaluated on a held-out test set (30%) using the area under the receiver operating characteristic curve (AUC) with bootstrap 95% confidence intervals, calibration metrics, and decision curve analysis. Internal validation comprised repeated 10-fold cross-validation and bootstrap optimism correction (1000 resamples). **Results:** Of 422 patients, 203 (48.1%) were diagnosed with MM. Random Forest, Gradient Boosting, and Ensemble Stacking showed similarly high discrimination on the test set (AUCs 0.950–0.955). Total protein (multivariable-adjusted OR: 11.95), calcium (OR: 9.62), hemoglobin (OR: 4.34), and albumin (OR: 0.16) were the strongest independent predictors. Ensemble Stacking showed the best calibration (Brier score 0.084; calibration slope 1.14). At a ≥95% sensitivity threshold, Random Forest maintained 77.3% specificity. Risk stratification effectively separated patients, with observed MM rates ranging from 2.4% (very low risk) to 97.2% (very high risk). **Conclusions:** Machine learning models using routine laboratory parameters achieved excellent diagnostic performance for discriminating MM from its clinical mimickers in a real-world referral cohort. Following further development with larger datasets and external validation, these models could support triage and prioritization of patients already under evaluation for suspected MM, particularly where access to specialized testing is delayed; use in lower-prevalence, first-contact settings would require recalibration and dedicated validation.

## 1. Introduction

Multiple myeloma (MM) is a clonal plasma cell malignancy characterized by bone marrow infiltration, monoclonal immunoglobulin production, and end-organ damage including anemia, hypercalcemia, renal dysfunction, and skeletal lesions [1,2]. With an annual incidence of approximately 6–7 per 100,000 individuals, MM accounts for 10% of hematologic malignancies and predominantly affects older adults (median age 69–70 years) [3,4,5,6]. Disparities in the timeliness of MM diagnosis have been reported across age groups and healthcare settings, with older patients and those presenting outside specialist pathways experiencing longer diagnostic intervals [7,8,9]. These clinical manifestations are often heterogeneous and non-specific, and patients may present with symptoms such as fatigue, bone pain, anemia, or renal insufficiency, which frequently overlap with common age-related conditions [10,11,12,13,14]. Early and accurate diagnosis of MM is critical because delayed identification is associated with increased morbidity and reduced survival, whereas timely intervention can mitigate organ damage and improve outcomes [7,8,9].

The current diagnostic gold standard for MM includes the detection of a clonal plasma cell population in the bone marrow and the demonstration of monoclonal protein via serum protein electrophoresis, immunofixation electrophoresis (IFE), or serum free light chain (FLC) assays. However, these specific assays are not universally available in all clinical settings, may incur significant cost, and often require specialist interpretation [9,15,16]. This limitation can lead to diagnostic delays, especially in resource-limited environments, primary care settings, and emergency departments where initial patient contact occurs. In contrast, routine laboratory tests (complete blood count [CBC], serum biochemistry, and inflammatory markers) are ubiquitously available and frequently constitute the first diagnostic evaluations.

Machine learning (ML) approaches have demonstrated promise in augmenting clinical decision-making across oncology and hematology by identifying complex, non-linear patterns within multimodal datasets that may be imperceptible to human clinicians [17,18,19,20]. Recent investigations have explored ML models to predict the risk of hematological malignancies based on routine health data, including applications in leukemia and lymphoma, underscoring their potential utility as screening and triage tools [21,22,23,24]. However, to date, most studies have relied on large administrative or population-based datasets rather than directly addressing the clinical challenge of distinguishing MM from mimickers in patients presenting with similar symptoms in real-world diagnostic pathways.

In this study, we hypothesized that ML models trained on routinely collected laboratory parameters can accurately discriminate patients with MM from those with similar clinical presentations but without evidence of monoclonal pathology on specific assays. To address this, we developed and internally validated multiple ML algorithms using a retrospective cohort of patients referred for suspected MM. We evaluated their discriminatory performance, predictor importance, calibration, and potential clinical utility. Our findings provide proof of concept that routinely available laboratory data, when analyzed using ML methodologies, can accurately distinguish MM from clinically similar conditions, thereby supporting earlier identification and optimized diagnostic workflows within the referral pathway, particularly where access to specific assays is delayed.

## 2. Materials and Methods

### 2.1. Study Design and Population

This retrospective diagnostic prediction model development and internal validation study included consecutive patients referred to the Department of Hematology for suspected multiple myeloma at a tertiary care center. All patients who underwent diagnostic workup for multiple myeloma between 2017 and 2025 were eligible for inclusion. The diagnosis of multiple myeloma was established according to the International Myeloma Working Group (IMWG) criteria; the final diagnosis was adjudicated by the treating hematologists on the basis of the complete diagnostic workup. The diagnostic workup included serum and urine protein electrophoresis, immunofixation, serum free light-chain assay, and bone marrow examination as clinically indicated. Because this was a retrospective study, the treating haematologists who adjudicated the final diagnosis were not blinded to the routine laboratory values that serve as model predictors; this is consistent with the intended clinical workflow, in which the same laboratory data would be available at the point of decision. Patients with incomplete diagnostic data or those who were lost to follow-up before definitive diagnosis were excluded.

The study was approved by the Institutional Ethics Committee, and the requirement for informed consent was waived due to the retrospective nature of the study. The study is reported in accordance with the TRIPOD+AI statement for prediction model studies involving machine learning.

### 2.2. Data Collection

Clinical and laboratory data were extracted from electronic medical records at the time of initial hematology consultation. Demographic variables included age and sex. Laboratory parameters included complete blood count (white blood cell count [WBC], red blood cell count [RBC], hemoglobin, platelet count, and mean corpuscular volume [MCV]) and serum biochemistry (creatinine, uric acid, total protein, albumin, lactate dehydrogenase [LDH], calcium, C-reactive protein [CRP], and erythrocyte sedimentation rate [ESR]). All predictor values were obtained at the time of initial hematology consultation, before the initiation of any myeloma-directed therapy and before any myeloma-specific diagnostic testing (serum and urine protein electrophoresis, immunofixation, serum free light chain assay, or bone marrow examination) was performed. The 15 predictor variables were selected on the basis of (a) routine availability at the point of haematology referral, (b) documented associations with multiple myeloma in the literature, and (c) data completeness (>80% non-missing values). Variables such as quantitative immunoglobulin levels (IgG, IgA, IgM), β2-microglobulin, and serum free light-chain concentrations were not routinely measured prior to the haematology referral and were therefore unavailable for a model intended to operate at the referral time point. Family history of myeloma and neuropathy history were not systematically documented in the electronic records and could not be reliably extracted. Including immunoglobulin or free light-chain values could improve discrimination but would change the model’s intended use from a routine-laboratory triage tool to a post-workup classifier.

### 2.3. Statistical Analysis

Continuous variables were assessed for normality using the Shapiro–Wilk test. Variables following a normal distribution are expressed as mean ± standard deviation and were compared using the independent samples *t*-test. Non-normally distributed variables are expressed as median with interquartile range (IQR) and were compared using the Mann–Whitney U test. Categorical variables are presented as frequencies and percentages and were compared using the Chi-square test or Fisher’s exact test, as appropriate.

Multicollinearity was assessed using the variance inflation factor (VIF), with VIF values greater than 10 considered indicative of problematic collinearity. Logistic regression was used both to identify potential predictors of multiple myeloma and as an interpretable statistical benchmark against which the machine learning models were compared. Univariable logistic regression analysis was performed first. All variables were then included simultaneously in a single multivariable logistic regression model without stepwise selection; predictors with *p* < 0.05 in this full model were described as independently associated with myeloma, and the full model is reported in Supplementary Table S1. Results are reported as odds ratios (ORs) with 95% confidence intervals (CIs). Continuous variables were standardized (z-score transformation) prior to logistic regression analysis to allow comparison of effect sizes. No formal a priori sample size calculation was performed; all eligible patients from the study period were included. With 203 outcome events and 15 candidate predictors, the events-per-variable ratio (approximately 13.5) exceeds the conventional minimum of 10 for the regression analyses, although it remains modest for flexible machine learning methods, as acknowledged in the Limitations.

### 2.4. Machine Learning Model Development

The dataset was randomly split into training (70%) and test (30%) sets using stratified sampling to maintain the proportion of myeloma cases in both sets. To prevent data leakage, all preprocessing steps were encapsulated within a single modelling pipeline (imputation; standardisation; classifier), ensuring that median imputation parameters and scaling parameters were fitted exclusively on training-fold data and applied to held-out folds in every cross-validation and bootstrap iteration. The learned preprocessing parameters from the full training set were then applied to the test set for final evaluation. Given the near-balanced outcome distribution (48.1% MM), no class imbalance correction methods (e.g., resampling or synthetic oversampling) were applied.

Multiple machine learning algorithms were trained and evaluated: (1) Logistic Regression with L1 regularization (LASSO), L2 regularization (Ridge), and standard maximum likelihood estimation; (2) Random Forest with hyperparameter optimization for number of trees, maximum depth, minimum samples for split, and minimum samples per leaf; (3) Gradient Boosting with tuning of learning rate, number of estimators, maximum depth, and subsample ratio; (4) Support Vector Machine (SVM) with radial basis function kernel and optimization of regularization parameter C and kernel coefficient gamma; and (5) Multilayer Perceptron Neural Network with optimization of hidden layer architecture and regularization parameter.

Hyperparameter optimization was performed using 5-fold cross-validation with grid search, selecting parameters that maximized the area under the receiver operating characteristic curve (AUC-ROC). Two ensemble methods were also developed: (1) Weighted Average Ensemble using AUC-based weights derived from training set performance and (2) Stacking Ensemble using a logistic regression meta-learner trained on cross-validated predictions from base models. Final hyperparameter values are provided in Supplementary Table S5.

### 2.5. Model Evaluation and Internal Validation

Primary model evaluation was performed on the held-out test set (30%) to reflect out-of-sample performance after all preprocessing and tuning were finalized using training data only. Model discrimination was assessed using the AUC-ROC, with 95% confidence intervals calculated using 1000 bootstrap resamples. Optimal classification thresholds were determined by maximizing Youden’s index (sensitivity + specificity − 1) on the training-set predictions and were then locked before application to the held-out test set. At optimal thresholds, sensitivity, specificity, positive predictive value (PPV), negative predictive value (NPV), accuracy, and F1 score were calculated. Bootstrap 95% confidence intervals (2000 resamples with replacement from the test set) were calculated for sensitivity, specificity, PPV, and NPV at each locked threshold. Additionally, thresholds achieving ≥90% and ≥95% sensitivity were identified to evaluate model performance in a screening context where minimizing false negatives is prioritized.

Model calibration was evaluated using multiple metrics: (1) Brier score, measuring the mean squared difference between predicted probabilities and observed outcomes; (2) calibration intercept (calibration-in-the-large), with values close to 0 indicating good calibration; and (3) calibration slope, with values close to 1 indicating good calibration. Bootstrap resampling (1000 samples) was used to calculate 95% confidence intervals for calibration metrics. Calibration curves were generated by plotting observed event rates against predicted probabilities.

Internal validation was performed using two approaches: (1) 10-fold cross-validation with 10 repetitions (100 total iterations) to assess model stability across different data partitions and (2) bootstrap resampling (1000 samples) to calculate optimism-corrected AUC values. For each bootstrap sample, a model was trained on the bootstrap sample and evaluated on both the bootstrap sample and the original dataset to estimate the optimism (difference between apparent and test performance). The entire modelling pipeline, including median imputation, standardisation, and model fitting, was repeated de novo within each bootstrap resample so that no preprocessing parameters leaked from the original training set into the optimism estimate.

### 2.6. Simple Rule Benchmarks

To evaluate whether the ML models add discrimination beyond readily available clinical rules, three benchmarks were evaluated on the same test-set patients: (1) the globulin gap (total protein minus albumin) as a single continuous predictor; (2) total protein alone as a single continuous predictor; and (3) an any-CRAB rule (calcium > 10.5 mg/dL, creatinine > 2 mg/dL, or haemoglobin < 10 g/dL) as a binary classifier. Benchmark AUCs with bootstrap 95% confidence intervals (2000 resamples) are reported alongside the ML model results. Paired bootstrap AUC differences (2000 resamples with replacement from the same test-set patients) tested whether each ML model achieved significantly higher discrimination than each benchmark, with significance defined by the 95% CI of the difference excluding zero.

### 2.7. CRAB Criterion Circularity Assessment

Calcium, creatinine, and haemoglobin are used both as model predictors and as components of the IMWG CRAB criteria that define myeloma-related organ damage. To assess whether the models’ discrimination partly reflects reconstruction of the reference standard, we performed two analyses: (1) a CRAB audit reporting the proportion of myeloma cases meeting each myeloma-defining event (hypercalcaemia [Ca > 10.5 mg/dL], renal impairment [Cr > 2 mg/dL], and anaemia [Hgb < 10 g/dL]) and (2) a sensitivity analysis in which logistic regression, Random Forest, and Ensemble Stacking models were retrained after excluding calcium, creatinine, and haemoglobin from the predictor set. The remaining variables, primarily total protein and albumin as surrogates for monoclonal protein burden, were used to quantify how much discrimination is independent of the CRAB criteria.

### 2.8. Clinical Utility Assessment

Decision curve analysis (DCA) was performed to assess the clinical utility of prediction models across a range of threshold probabilities. Net benefit was calculated as the difference between the proportion of true positives and the proportion of false positives weighted by the odds of the threshold probability. Because all patients were already referred to haematology, the clinical action evaluated by DCA was defined as prioritising definitive myeloma workup (serum/urine protein electrophoresis, serum free light-chain assay, and bone marrow examination) within the referral queue. The net benefit of each model was compared against the strategies of ordering definitive workup for all referred patients and ordering it for none.

Clinical utility was further evaluated by stratifying patients into five risk categories based on predicted probabilities (<20%, 20–40%, 40–60%, 60–80%, and >80%) and calculating observed myeloma rates within each category. As an additional exploratory analysis, risk stratification was repeated using the Ensemble Stacking model with three predefined cutoffs (<20%, 20–50%, >50%) chosen to separate clinically actionable risk groups. Wilson score confidence intervals were calculated for the observed rates in each risk category.

### 2.9. Model Interpretability

Variable importance was assessed using model-specific methods: mean decrease in Gini impurity for Random Forest and feature importance scores for Gradient Boosting. For enhanced interpretability, SHapley Additive exPlanations (SHAP) analysis was performed on the Gradient Boosting model to quantify the contribution of each feature to individual predictions. SHAP values provide both the magnitude and direction of feature effects, enabling a more nuanced understanding of model behavior.

### 2.10. Software

All statistical analyses and machine learning model development were performed using Python version 3.10 with the following libraries: pandas and numpy for data manipulation, scipy for statistical tests, scikit-learn for machine learning algorithms, statsmodels for logistic regression analysis, and SHAP for model interpretability. A two-sided *p*-value < 0.05 was considered statistically significant.

## 3. Results

### 3.1. Study Population and Baseline Characteristics

**Figure 1:**
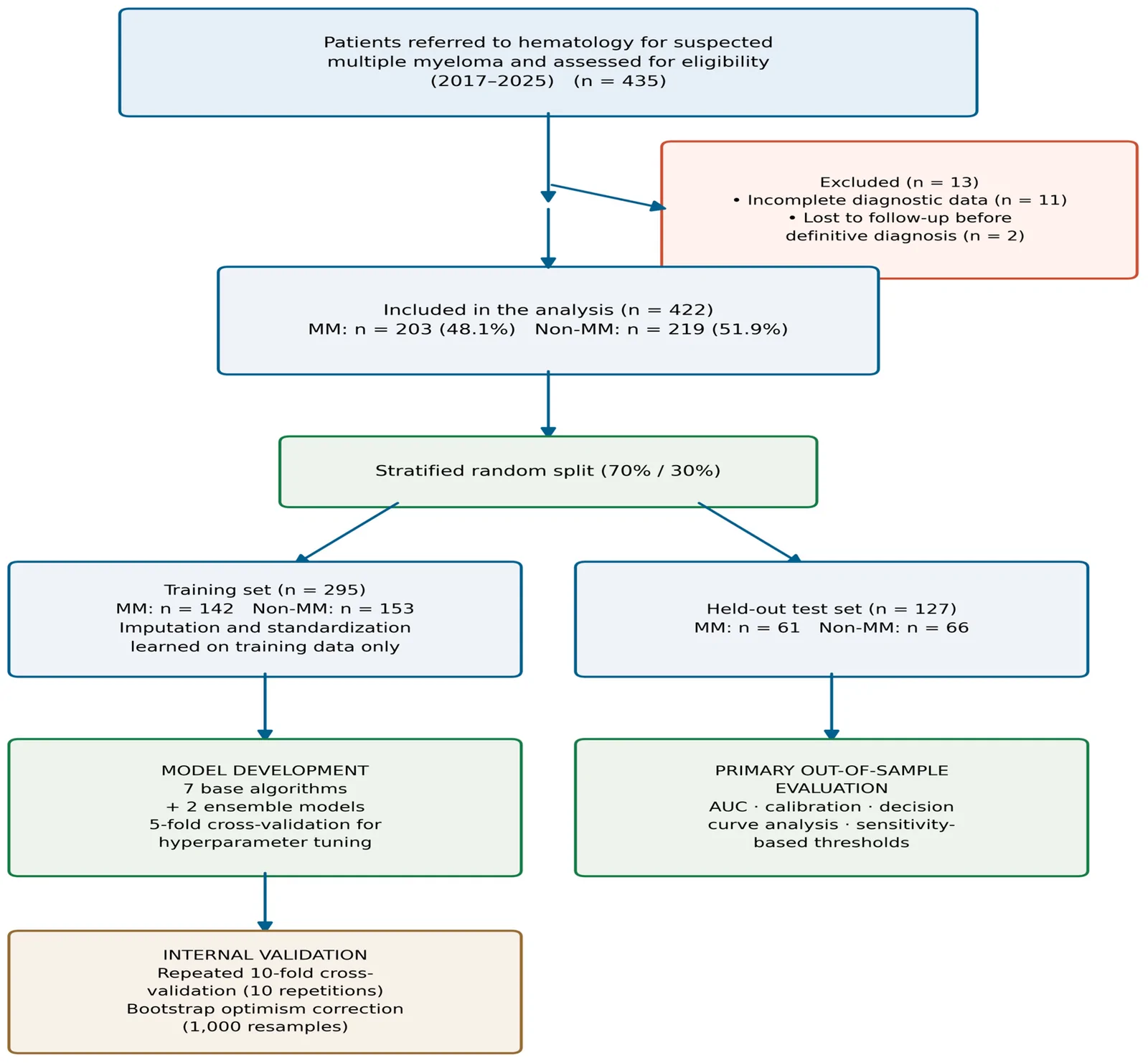
Study flow diagram. Flow of participants from eligibility assessment through exclusion, stratified allocation to training and held-out test sets, model development, out-of-sample evaluation on the held-out test set, and internal validation.

Of 435 patients referred to hematology for suspected multiple myeloma and assessed for eligibility between 2017 and 2025, 13 were excluded (incomplete diagnostic data, *n* = 11; loss to follow-up before definitive diagnosis, *n* = 2), leaving 422 patients for analysis (Figure 1). Of these, 203 (48.1%) were diagnosed with multiple myeloma, while 219 (51.9%) were classified as non-myeloma cases. The baseline characteristics of the study population stratified by myeloma status are presented in Table 1.

The 219 non-myeloma patients comprised individuals referred for suspected multiple myeloma in whom the diagnosis was excluded after complete work-up; their clinical presentations and final diagnoses are detailed in Table 2. Anemia was the most frequent referral trigger, recorded as unspecified/multifactorial anemia (53.4%), anemia of chronic kidney disease (19.2%), or iron deficiency anemia (17.8%). Chronic kidney disease/renal impairment was documented in 21.9% and solid malignancies in 11.0%. No cases of MGUS, smoldering multiple myeloma, solitary plasmacytoma, or AL amyloidosis were present among the controls; the single patient with amyloidosis had non-plasma-cell (cardiac/AA-type) disease. Clonal or malignant hematological conditions retained in the control group included myelodysplastic syndrome, primary myelofibrosis, chronic myeloid leukemia, and B-cell lymphomas. Hypercalcemia was present in only one non-myeloma patient (*n* = 1). Because categories are not mutually exclusive, percentages sum to more than 100%.

### 3.2. Collinearity Assessment

Variance inflation factor analysis confirmed the absence of problematic multicollinearity among the included variables. All VIF values were below 2.0, with the highest values observed for albumin (VIF = 1.74), uric acid (VIF = 1.66), and ESR (VIF = 1.62).

### 3.3. Logistic Regression Results

Univariable logistic regression analysis identified several significant predictors of myeloma (Table 3). The strongest positive predictors included total protein (OR: 5.92; 95% CI: 3.62–9.69; *p* < 0.001), calcium (OR: 2.59; 95% CI: 1.77–3.80; *p* < 0.001), creatinine (OR: 1.93; 95% CI: 1.41–2.65; *p* < 0.001), hemoglobin (OR: 1.88; 95% CI: 1.43–2.45; *p* < 0.001), and ESR (OR: 1.85; 95% CI: 1.44–2.38; *p* < 0.001). Significant negative predictors included platelet count (OR: 0.43; 95% CI: 0.32–0.59; *p* < 0.001) and albumin (OR: 0.55; 95% CI: 0.42–0.71; *p* < 0.001).

In multivariable logistic regression analysis (Table 3 and Table S1), seven variables remained independently associated with myeloma diagnosis. Total protein was the strongest independent predictor (OR: 11.95; 95% CI: 4.22–33.88; *p* < 0.001), followed by calcium (OR: 9.62; 95% CI: 3.84–24.12; *p* < 0.001), hemoglobin (OR: 4.34; 95% CI: 2.36–8.01; *p* < 0.001), and creatinine (OR: 2.33; 95% CI: 1.35–4.04; *p* = 0.003). Albumin showed a strong inverse association (OR: 0.16; 95% CI: 0.08–0.33; *p* < 0.001). Platelet count (OR: 0.46; 95% CI: 0.26–0.81; *p* = 0.007) and female sex (OR: 0.60; 95% CI: 0.38–0.93; *p* = 0.021) also showed significant negative associations.

### 3.4. Machine Learning Model Performance

**Figure 2:**
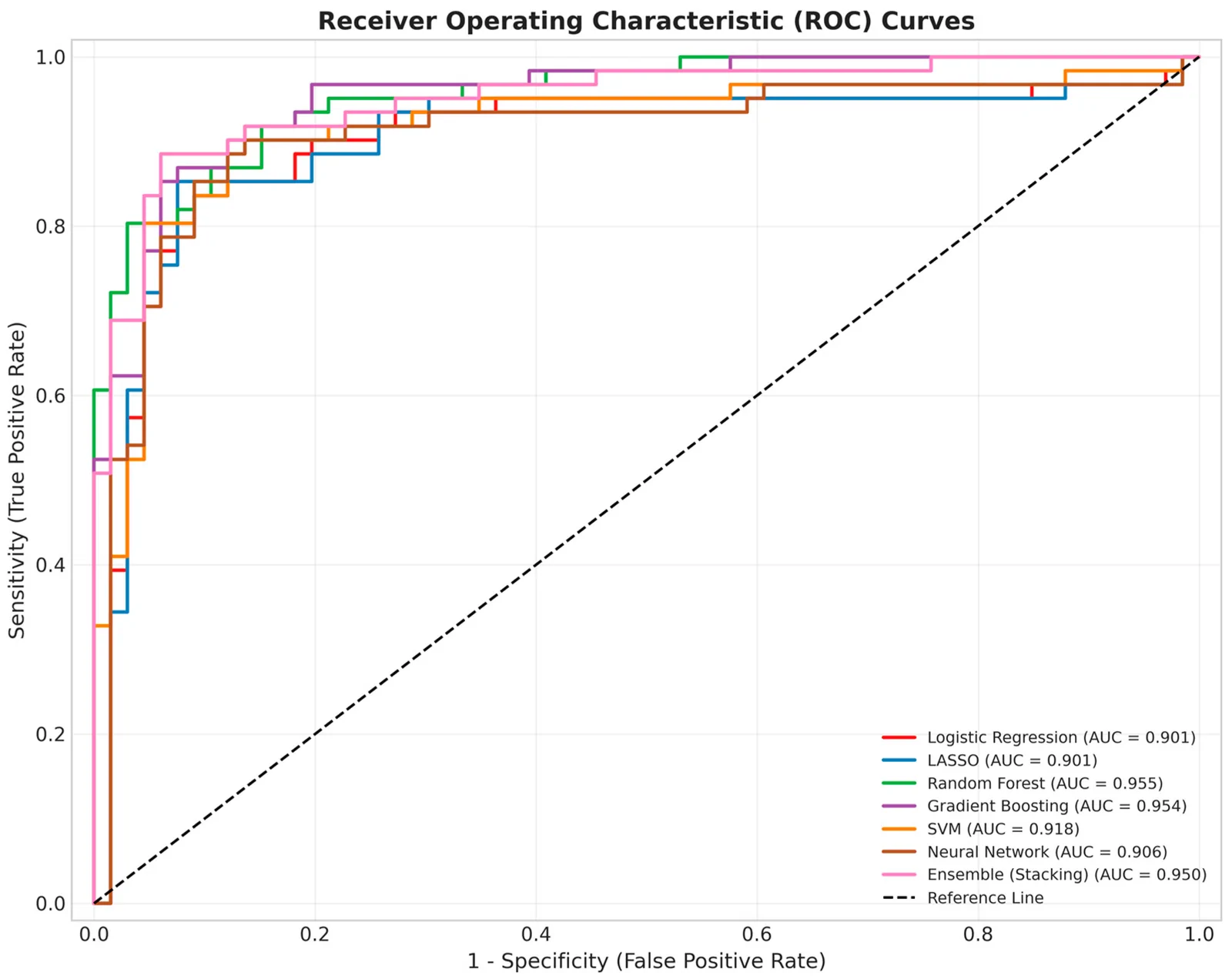
Receiver Operating Characteristic (ROC) curves for selected prediction models on the test set. Ensemble (Weighted) and Ridge regression are omitted for visual clarity; performance of all nine models is reported in Table 4.

The dataset was split into training (*n* = 295, 70%; 142 MM) and test (*n* = 127, 30%; 61 MM) sets. Nine machine learning models were evaluated on the test set (Table 4, Figure 2). All models demonstrated high discriminative ability with AUC values exceeding 0.90 on the test set.

Random Forest showed the highest point-estimate AUC (0.955; 95% CI: 0.921–0.981), followed by Gradient Boosting (AUC: 0.954; 95% CI: 0.918–0.984) and Ensemble Stacking (AUC: 0.950; 95% CI: 0.905–0.980). Confidence intervals overlapped substantially across all models; the ranking is therefore descriptive, and the top-performing models should be regarded as having statistically indistinguishable discrimination in this sample. At optimal cutoff thresholds determined by Youden’s index, Ensemble Stacking demonstrated a sensitivity of 88.5% and specificity of 93.9%, with a PPV of 93.1% and NPV of 89.9%.

As benchmarks, the globulin gap (total protein minus albumin) achieved an AUC of 0.769 (95% CI 0.683–0.846), total protein alone achieved 0.789 (95% CI 0.712–0.859), and the any-CRAB rule achieved 0.321 (95% CI 0.263–0.381) on the same 127 test-set patients. Paired bootstrap testing (Table 5) showed that Ensemble Stacking significantly outperformed all three benchmarks: ΔAUC = 0.194 (95% CI 0.111–0.281) versus the globulin gap, 0.176 (95% CI 0.101–0.259) versus total protein alone, and 0.642 (95% CI 0.571–0.717) versus the any-CRAB rule. The comparison of Ensemble Stacking against logistic regression yielded a smaller but statistically significant improvement (ΔAUC = 0.023, 95% CI 0.006–0.046).

### 3.5. CRAB Criterion Circularity Analysis

Among 203 myeloma patients, 138 (68.0%) met at least one CRAB criterion: 106 (52.2%) had anaemia (Hgb < 10 g/dL), 63 (31.0%) had renal impairment (Cr > 2 mg/dL), and 45 (22.2%) had hypercalcaemia (Ca > 10.5 mg/dL). In the sensitivity analysis excluding calcium, creatinine, and haemoglobin from the predictor set, logistic regression AUC was 0.935 (vs. 0.901 with full predictors), Random Forest AUC was 0.946 (vs. 0.955), and Ensemble Stacking AUC was 0.946 (vs. 0.950) (Table 6). The modest reductions (ΔAUC 0.004–0.009) indicate that discrimination is primarily driven by total protein and albumin, surrogates for monoclonal protein burden, rather than by reconstruction of the CRAB diagnostic criteria.

### 3.6. False-Negative Audit by Globulin Gap Level

To evaluate whether the model fails disproportionately in cases with a low globulin gap, we stratified all 61 myeloma cases in the test set by globulin gap level (Table 7). Among cases with a low globulin gap (≤3.5 g/dL, *n* = 20), the Ensemble Stacking model achieved a sensitivity of 0.800, with 4 of 20 cases missed. In the moderate group (3.5–5.0 g/dL, *n* = 13), sensitivity was 0.846 with 2 cases missed. All 28 cases with a high globulin gap (>5.0 g/dL) were correctly identified (sensitivity 1.000). These findings confirm that the model’s discriminative ability is concentrated in cases with elevated globulin and suggest reduced sensitivity for disease subtypes with low or absent monoclonal protein elevation.

### 3.7. Prevalence-Adjusted Predictive Values

Using the Ensemble Stacking model’s sensitivity (88.5%) and specificity (93.9%) at its Youden-index threshold, PPV and NPV were projected across five prevalence scenarios to illustrate how performance would shift in lower-prevalence settings (Table 8). At the cohort prevalence of 48.1%, the model achieved a PPV of 0.931 and NPV of 0.899. At a prevalence of 5%, representative of a general haematology outpatient setting, the projected PPV dropped to 0.434 while the NPV remained 0.994, indicating that the model retains strong rule-out capability but generates a substantial false-positive burden in lower-prevalence populations.

**Table 8:** Prevalence-Adjusted PPV and NPV (Ensemble Stacking).

Myeloma Prevalence	Projected PPV	Projected NPV
1% (general population)	0.127	0.999
5% (general haem outpatient)	0.434	0.994
10% (targeted referral)	0.618	0.987
20% (enriched referral)	0.784	0.970
48.1% (present cohort)	0.931	0.899

### 3.8. Internal Validation

Cross-validation results demonstrated robust model performance across different data partitions (Table S2). Random Forest achieved the highest mean AUC in repeated 10-fold cross-validation (0.950 ± 0.032), followed by SVM (0.948 ± 0.036) and Logistic Regression (0.942 ± 0.039).

Bootstrap internal validation with 1000 resamples (Table S3) revealed low estimated optimism across all evaluated models. Random Forest showed an apparent AUC of 1.000 with an estimated optimism of 0.011, yielding an optimism-corrected AUC of 0.989 (95% CI: 0.982–0.995). These optimism-corrected estimates should be interpreted with caution, particularly for Random Forest: tree ensembles reproduce the training data almost perfectly, so their apparent performance approaches unity by construction and the resulting optimism estimate is correspondingly compressed. Accordingly, the held-out test set results reported above, rather than the optimism-corrected values, are regarded as the primary performance estimates of this study. Gradient Boosting demonstrated similar results with a corrected AUC of 0.984 (95% CI: 0.971–0.995), and Ensemble Stacking showed a corrected AUC of 0.978 (95% CI: 0.969–0.985). The apparent AUC of 1.000 for tree-based models reflects their capacity to fit training data perfectly; the corrected estimates should therefore be interpreted alongside the more conservative held-out test set and cross-validation results.

### 3.9. Model Calibration

**Figure 3:**
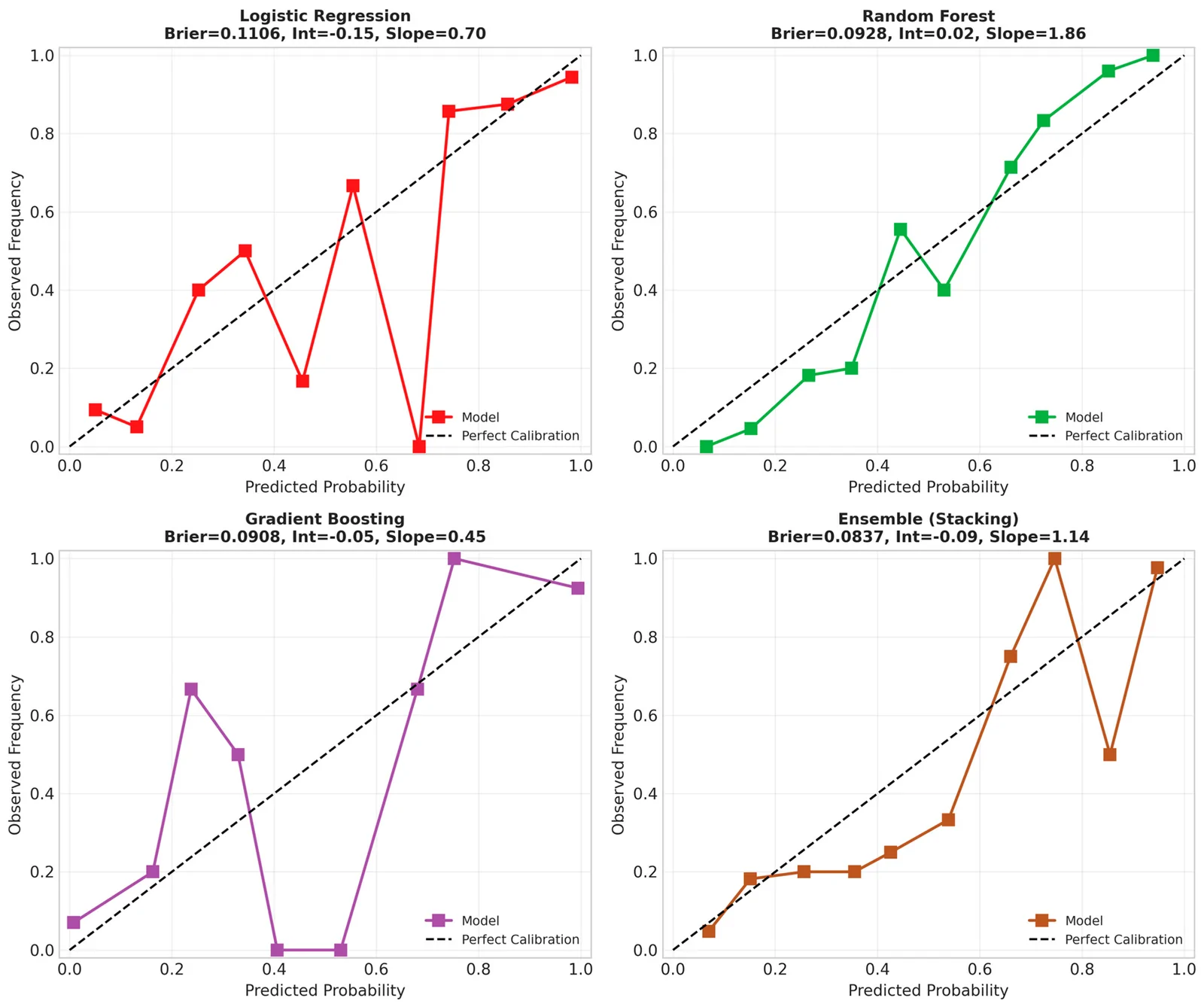
Calibration curves for selected models.

Calibration performance was assessed using Brier score, calibration intercept, and calibration slope with bootstrap 95% confidence intervals (Table S4, Figure 3). The Ensemble Stacking model demonstrated the best overall calibration, with the lowest Brier score (0.084), a calibration intercept close to zero (−0.09; 95% CI: −0.77 to 0.56), and a calibration slope close to one (1.14; 95% CI: 0.87–1.67). Random Forest showed strong discrimination but imperfect calibration, with a slope of 1.80 (95% CI: 1.34–2.69), indicating under-dispersed, overly conservative probability estimates that did not spread sufficiently across the observed risk gradient. Gradient Boosting exhibited a slope of 0.60 (95% CI: 0.45–0.93), indicating overly extreme predictions consistent with residual overfitting and a need for shrinkage or recalibration. Logistic Regression showed a slope of 0.70 (95% CI: 0.46–1.19), likewise suggesting somewhat too-extreme predictions. Given the statistically overlapping discrimination among the top-performing models, the superior calibration of Ensemble Stacking supports its use as the primary model where predicted probabilities are interpreted directly.

### 3.10. Sensitivity-Based Threshold Analysis

For clinical application as a screening tool where minimizing missed diagnoses is prioritized, we evaluated model performance at thresholds achieving ≥90% and ≥95% sensitivity (Table S5). At the ≥95% sensitivity threshold, Random Forest maintained the best balance with a specificity of 77.3%, PPV of 79.5%, and NPV of 94.4%. Ensemble Stacking achieved 95.1% sensitivity with 72.7% specificity, PPV of 76.3%, and NPV of 94.1%. These results suggest that the models can effectively identify patients requiring hematology referral while maintaining acceptable specificity.

### 3.11. Clinical Utility and Decision Curve Analysis

**Figure 4:**
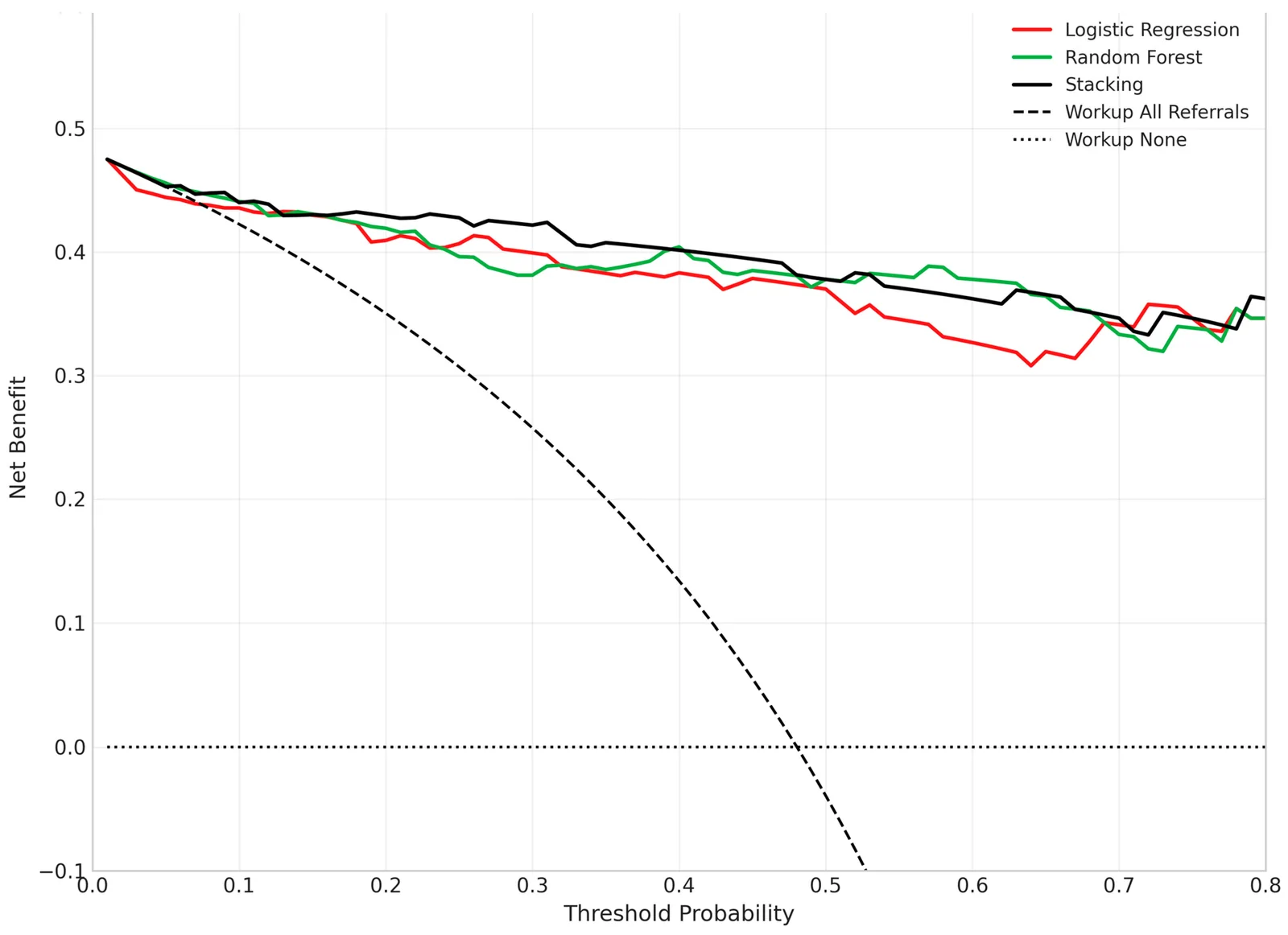
Decision Curve Analysis showing net benefit across threshold probabilities for prioritising definitive myeloma workup (SPEP, UPEP, sFLC, bone marrow assessment) within the haematology referral queue. The “Workup All Referrals” line represents ordering definitive workup for every referred patient; the “Workup None” line represents a strategy of no further investigation.

Decision curve analysis demonstrated that all prediction models provided net benefit superior to the default strategies of treating all or treating none across a wide range of threshold probabilities (Figure 4). For threshold probabilities between 10% and 70%, all models showed positive net benefit, indicating clinical utility for prioritizing definitive myeloma diagnostic work-up. Random Forest and Ensemble Stacking consistently showed the highest net benefit across most threshold ranges.

### 3.12. Variable Importance and Shap Analysis

Variable importance analysis consistently identified total protein as the most important predictor across all modeling approaches (Figure S1). SHAP analysis was applied to the Gradient Boosting model as a representative high-performing tree-based learner; the analysis (Figure S2) confirmed these findings and provided additional insights: higher total protein values strongly increased myeloma probability, while higher albumin levels decreased myeloma probability. Elevated hemoglobin, calcium, and creatinine values were associated with increased myeloma risk. The SHAP summary plot revealed non-linear relationships and potential interactions between features.

### 3.13. Clinical Risk Stratification

**Figure 5:**
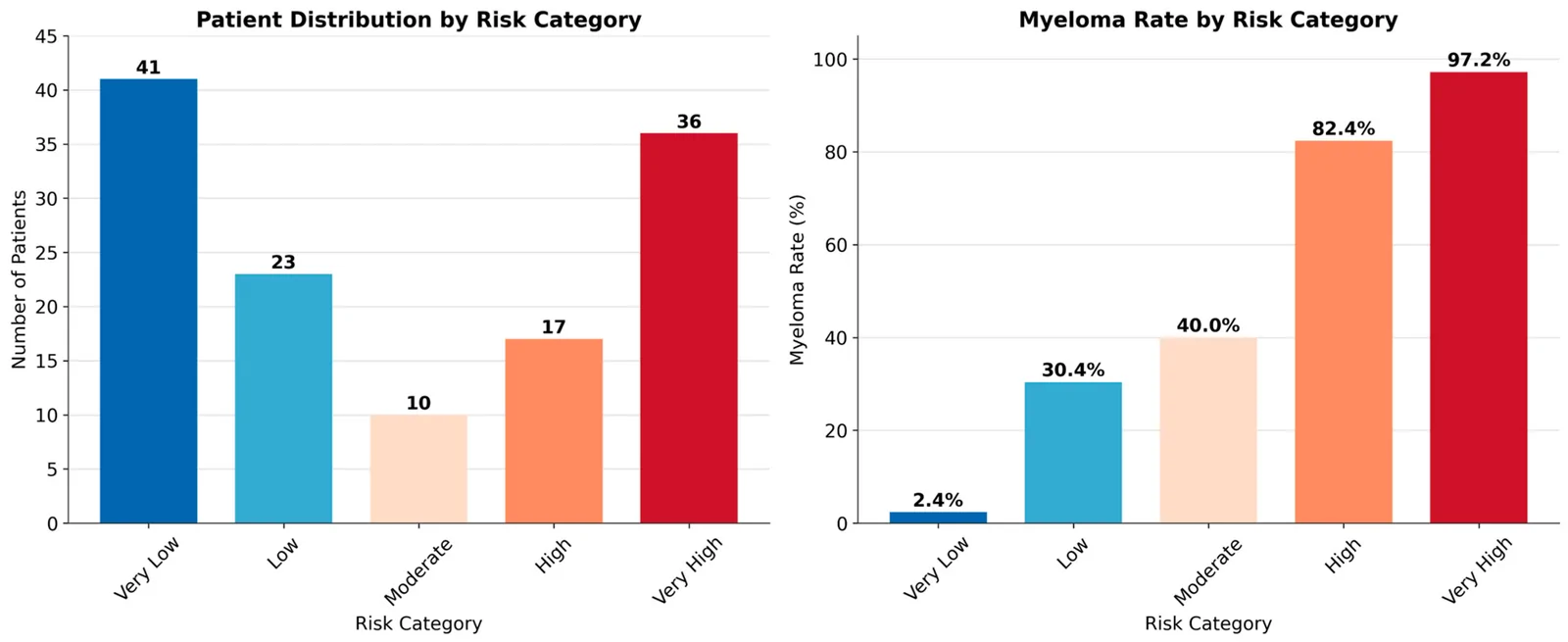
Clinical risk stratification based on Random Forest model predictions.

Based on Random Forest model predictions, patients in the test set were stratified into five risk categories with Wilson score 95% confidence intervals (Table S6, Figure 5). The very low-risk category (<20% predicted probability) included 41 patients with an observed myeloma rate of only 2.4% (95% CI: 0.4–12.6%). The low-risk category (20–40%) included 23 patients with a 30.4% myeloma rate (95% CI: 15.6–50.9%). The moderate-risk category (40–60%) included 10 patients with a 40.0% myeloma rate (95% CI: 16.8–68.7%). The high-risk category (60–80%) included 17 patients with an 82.4% myeloma rate (95% CI: 59.0–93.8%). Finally, the very high-risk category (>80%) included 36 patients with an observed myeloma rate of 97.2% (95% CI: 85.8–99.5%). Separation was clear at the extremes of predicted risk, whereas estimates in the intermediate categories were imprecise owing to small cell sizes, as reflected in their wide confidence intervals.

Because the calibration slope of Random Forest (1.80) indicated under-dispersed probability estimates, we performed an additional exploratory risk stratification using the Ensemble Stacking model, which showed better calibration (slope 1.14, Brier score 0.084). Three predefined risk groups were defined using cutoffs at 20% and 50% predicted probabilities. Among 55 patients classified as low risk (<20%), 3 (5.5%) had myeloma. Among 14 intermediate-risk patients (20–50%), 5 (35.7%) had myeloma. Among 58 high-risk patients (>50%), 53 (91.4%) had myeloma. These three-tier groups showed clear separation and may offer a more clinically actionable framework, although they require external validation before adoption.

## 4. Discussion

In this study, we developed and internally validated machine learning (ML) models for discriminating multiple myeloma (MM) from clinically similar conditions using exclusively routine laboratory parameters. Because the cohort comprised patients already referred to hematology with clinical suspicion of MM, our findings pertain to discrimination within a referral population—patients awaiting or lacking access to specific monoclonal-protein testing—rather than to unselected screening; predictive values are anchored to the high outcome prevalence (48.1%) of this setting and would differ substantially at lower prevalence. Within this frame, Random Forest, Gradient Boosting, and ensemble models demonstrated high discriminative performance, with AUC values exceeding 0.95, sensitivities of 80–90%, and specificities of 86–97% on the held-out test set. Total protein, calcium, hemoglobin, albumin, and platelet count emerged as the most influential independent predictors, although the directions of some associations—most notably hemoglobin—reflected the composition of the comparator population rather than classical disease biology, as discussed below. Risk stratification achieved clear separation at the extremes of predicted risk, with observed MM rates of 2.4% in the very low-risk and 97.2% in the very high-risk group, although estimates in the intermediate categories were imprecise; this pattern supports the potential utility of these models for triage and prioritization within the referral pathway.

Total protein (OR: 11.95) and albumin (OR: 0.16) showed the strongest predictive value, consistent with the biological hallmark of MM—monoclonal immunoglobulin overproduction by clonal plasma cells [2,25]. The combination of elevated total protein with reduced albumin reflects the albumin–globulin gap, which may indirectly signal monoclonal gammopathy even in the absence of serum protein electrophoresis [26,27]. In the present study, the globulin gap alone achieved an AUC of 0.769 on the test set, confirming its value as a simple, universally available discriminator. However, paired bootstrap analysis demonstrated that Ensemble Stacking provided a statistically significant improvement (ΔAUC = 0.194; 95% CI 0.111–0.281) over the globulin gap by capturing non-linear interactions among multiple laboratory parameters. Conversely, the improvement of Ensemble Stacking over standard logistic regression was small (ΔAUC = 0.023), suggesting that much of the ML advantage lies in combining protein-based signals with other routine markers rather than in the algorithmic complexity per se. In settings where computational infrastructure is limited, logistic regression or even the globulin gap alone may serve as pragmatic alternatives. These findings underscore that biologically meaningful disease signatures can be detected through routine laboratory patterns without reliance on specialized assays.

Hypercalcemia (OR: 9.62) provided substantial diagnostic information despite only modest absolute differences between groups (median 9.4 vs. 8.9 mg/dL). Although overt hypercalcemia was uncommon in our cohort, calcium remained among the most informative predictors in the ML models. This finding likely reflects referral patterns and selection bias rather than purely biological mechanisms. Patients with isolated hypercalcemia are typically evaluated for primary hyperparathyroidism, solid malignancies, or endocrine disorders and may not reach hematology unless other CRAB features (anemia, renal dysfunction, bone disease) are present [28,29,30]. Consequently, hypercalcemia was rare in our control group (comprising patients referred for suspected MM but ultimately ruled out), thereby generating a strong discriminatory signal for the models.

In contrast, MM patients more frequently showed hypercalcemia alongside other disease manifestations, consistent with osteoclast activation via RANKL, IL-6, and macrophage inflammatory protein-1α [31,32]. Rather than relying on fixed diagnostic thresholds, the ML models appear to capture relative differences, distributional patterns, and interactions between calcium and other routine parameters. Consequently, even small numerical differences gained diagnostic relevance when interpreted in combination with total protein, albumin, and renal function. This illustrates a key advantage of ML-based approaches: the ability to detect biologically meaningful yet clinically subtle signals that are typically overlooked in conventional threshold-based clinical assessment.

A notable finding was that several traditionally emphasized MM-related parameters showed unexpected discriminative patterns that reflect the clinical characteristics of the comparator population rather than contradictions of MM biology. Most striking was the positive association between hemoglobin and myeloma diagnosis (multivariable OR: 4.34), which appears paradoxical given that anemia is a hallmark feature of MM [33]. This reflects the non-myeloma group’s composition: referred for suspected MM, these patients frequently had conditions causing significant anemia—iron deficiency, chronic kidney disease, chronic inflammation, and other hematological disorders [34,35,36]. Consequently, the non-myeloma group exhibited more pronounced anemia (median hemoglobin 9.2 g/dL) compared to MM patients (median 9.9 g/dL). Similarly, the inverse association for platelet count (OR: 0.46) likely reflects reactive thrombocytosis in the non-myeloma group from chronic inflammation or iron deficiency, whereas MM patients more often had normal or reduced counts [37,38,39,40,41,42]. Creatinine contributed only modestly (OR: 2.33) because renal dysfunction was prevalent in both groups. Importantly, the non-myeloma group was not enriched by excluding difficult near-neighbor diagnoses: no cases of MGUS, smoldering myeloma, solitary plasmacytoma, or AL amyloidosis were present, whereas clonal and malignant hematological conditions—including myelodysplastic syndrome, primary myelofibrosis, chronic myeloid leukemia, and B-cell lymphomas—were retained as controls, representing genuinely challenging discriminations rather than trivially separable comparators. Hypercalcemia was essentially absent among non-myeloma patients (*n* = 1), indicating that the model’s discrimination is not attributable to a hypercalcemia imbalance between groups. Because calcium, creatinine, and haemoglobin also contribute to the IMWG CRAB criteria used to define myeloma-related organ damage, the possibility of criterion circularity was assessed. In a sensitivity analysis that excluded these three predictors, all models retained AUC values above 0.93 (reductions of 0.004–0.009), confirming that the models’ discrimination is primarily driven by monoclonal-protein surrogates (total protein and albumin) rather than by partial reconstruction of the CRAB diagnostic criteria. These observations underscore that biomarker utility is shaped by the comparator population: models trained on clinically realistic differential-diagnostic cohorts prioritize disease-specific signals (monoclonal protein production, altered bone metabolism) over non-specific features confounded by comorbidities. Similarly, the inverse association observed for age and the protective association for female sex likely reflect referral and spectrum effects within a hematology-suspected cohort, where non-myeloma differentials in older and female patients may be over-represented.

Recent studies have increasingly explored the use of artificial intelligence in MM detection using routine clinical data. Yao et al. reported ML models achieving AUC values approaching 0.99 in classifying plasma cell dyscrasias using common laboratory parameters [43]. Similarly, Li et al. developed a diagnostic model distinguishing MM from connective tissue diseases with abnormal immunoglobulin levels, achieving excellent discrimination (AUC 0.98) using variables including IgM, glomerular filtration rate, HDL, red cell distribution width, and thrombin time [44]. While impressive, these studies rely on immunological parameters (immunoglobulin levels, protein electrophoresis) that are not universally available and already represent disease-oriented testing. In contrast, our study deliberately restricted model inputs to universally available routine parameters, addressing a more challenging yet clinically relevant diagnostic scenario, with potential applicability in settings where access to specialized testing is limited, subject to the validation requirements discussed below.

Our ensemble models, particularly Random Forest and Gradient Boosting, achieved performance comparable to prior reports (AUC 0.90–0.95) while maintaining strict exclusion of specialized biomarkers, and internal validation with repeated cross-validation and bootstrap optimism correction supported the stability of these estimates. SHAP analysis further enhanced interpretability by quantifying individual feature contributions, addressing concerns regarding the “black box” nature of ML [45,46,47,48].

Decision curve analysis demonstrated net benefit compared to referring all or no patients across threshold probabilities of 10–70%. At ≥95% sensitivity, Random Forest maintained 77% specificity, identifying approximately three-quarters of non-MM patients while missing fewer than 5% of true cases. This performance profile is particularly relevant given that delayed diagnosis remains a major challenge in multiple myeloma. Previous studies have reported that the time between first symptom presentation and confirmed diagnosis often exceeds three months, with mean diagnostic intervals of approximately 100 days [49,50]. Such delays are associated with more advanced organ damage at presentation and worse clinical outcomes, underscoring the need for tools that can support earlier suspicion and prioritization for definitive diagnostic workup.

Because they rely solely on routine laboratory tests, the models could be embedded into electronic health record systems as automated decision-support tools that calculate MM probability once results become available, supporting triage and prioritization of patients already under evaluation for suspected MM—for example, by accelerating access to electrophoresis, free light chain testing, and bone marrow assessment for high-probability patients where such testing is delayed or capacity-limited. Extension to lower-prevalence, first-contact settings such as primary care or emergency departments [51] is an attractive but distinct use case that would require recalibration and dedicated external validation in those populations before any deployment. Importantly, these tools are intended to complement rather than replace clinical judgment; high-probability predictions should prompt definitive diagnostic workup rather than substitute for it. At implementation, predictions should be generated only when the required laboratory inputs are available and of adequate quality; when one or more inputs are missing, the model output should be suppressed rather than imputed at the point of care. Because the model would operate on automatically retrieved laboratory values, no specialised user interaction beyond standard clinical interpretation of the resulting probability would be required.

### 4.1. Limitations

This study has several limitations. First, its retrospective, single-center design introduces potential selection and information bias and limits generalizability. Additionally, the cohort represents patients referred for suspected myeloma, resulting in a high outcome prevalence (48.1%) and a clinically enriched spectrum; model performance and predictive values are therefore expected to differ in lower-prevalence settings such as primary care. Additionally, the random 70/30 train–test split does not capture temporal drift in clinical practice or referral patterns between 2017 and 2025; a temporal split would have been preferable but was not feasible given the sample size. Because the same routine parameters used as model inputs may also have influenced the original referral decision, a degree of incorporation bias cannot be excluded, and the models may partly recapitulate the clinical reasoning that generated the referral rather than a disease-specific signal alone. Furthermore, penalized and standard logistic regression achieved AUCs of 0.901, indicating that much of the discriminative signal is linear and concentrated in a small number of parameters; the incremental value of complex models over simple benchmarks was formally quantified through paired bootstrap AUC comparisons, demonstrating statistically significant but modest gains over logistic regression and more substantial gains over the globulin gap alone.

Second, although extensive internal validation was conducted using repeated cross-validation and bootstrap optimism correction, external validation in independent cohorts is lacking. As with most prediction models, performance may decline when applied to new populations. Some degree of residual overfitting, particularly in tree-based models, cannot be fully excluded, and probability calibration varied across algorithms; bootstrap optimism estimates may additionally be anti-conservative if the full model-selection process is not repeated within each resample. Third, the modest sample size and relatively small test set may limit the precision of performance estimates. With 203 cases and 15 predictors, the events-per-variable ratio (~13.5) is at the lower recommended boundary, contributing to uncertainty in some coefficient estimates and the wide confidence intervals seen for certain odds ratios. In addition, although age and sex were included as predictors, model performance was not evaluated separately within demographic subgroups, and fairness across such subgroups remains to be assessed in larger validation cohorts. Finally, the model relied exclusively on routinely available laboratory parameters and did not incorporate myeloma-specific biomarkers, imaging findings, or clinical symptoms. While this supports use as an early screening tool, it may limit discriminative ability compared with more comprehensive diagnostic models. The outcome was restricted to myeloma diagnosis and does not provide prognostic or staging information.

### 4.2. Future Directions

Future research should prioritize multicenter external and prospective validation to evaluate clinical impact, including effects on time to diagnosis and use of specialized testing. In the present revision, paired bootstrap comparisons demonstrated statistically significant improvements of the ML models over the globulin gap, total protein alone, and a CRAB-based rule; future work should extend this comparison to prospective settings where the clinical consequences of model-guided triage can be measured directly. Refinement through additional clinical variables, biomarkers such as β2-microglobulin, and longitudinal laboratory trends, together with implementation and health-economic studies, will be necessary for real-world deployment.

## 5. Conclusions

Machine learning models trained exclusively on routine laboratory parameters achieved high diagnostic performance for multiple myeloma in a real-world hematology referral cohort. Total protein, albumin, calcium, and platelet count provided the strongest discriminatory signals, with platelet count offering additional value by capturing reactive patterns common in non-malignant conditions. These findings support the potential of ML-based analysis of routine laboratory data as a practical clinical decision support tool for earlier MM recognition and optimized diagnostic workflows, particularly in settings where access to specialized testing may be delayed. External validation and prospective evaluation are required before clinical implementation.

## Figures and Tables

**Table 1:** Baseline Characteristics of Study Population.

Variable	Total (*n* = 422)	Myeloma (*n* = 203)	Non-Myeloma (*n* = 219)	*p*-Value
Demographics				
Age, years	71.0 (64.0–78.0)	69.0 (61.5–76.0)	73.0 (67.0–80.0)	<0.001
Female sex, *n* (%)	226 (53.6)	88 (43.3)	138 (63.0)	<0.001
Hematological Parameters				
WBC, ×10^9^/L	6.21 (4.77–7.95)	6.10 (4.68–7.90)	6.22 (4.86–8.00)	0.396
RBC, ×10^12^/L	3.3 (2.9–3.8)	3.3 (2.9–3.9)	3.4 (3.0–3.7)	0.752
Hemoglobin, g/dL	9.4 (8.4–10.0)	9.9 (8.6–11.5)	9.2 (8.4–9.7)	<0.001
Platelet, ×10^9^/L	236 (171–292)	218 (150–261)	260 (189–341)	<0.001
MCV, fL	90.2 (83.8–95.1)	92.4 (87.2–96.0)	87.5 (80.6–93.5)	<0.001
Biochemical Parameters				
Creatinine, mg/dL	1.1 (0.9–1.8)	1.2 (0.9–2.2)	1.1 (0.8–1.5)	0.002
Uric acid, mg/dL	6.3 (5.0–7.8)	6.8 (5.1–8.6)	6.2 (4.9–7.4)	0.002
Total protein, g/dL	7.2 (6.5–8.3)	8.3 (7.0–10.0)	6.7 (6.3–7.2)	<0.001
Albumin, g/dL	3.8 (3.2–4.1)	3.6 (3.2–4.0)	3.9 (3.4–4.2)	<0.001
LDH, U/L	196 (163–247)	193 (153–241)	199 (168–265)	0.038
Calcium, mg/dL	9.1 (8.6–9.7)	9.4 (8.9–10.5)	8.9 (8.6–9.4)	<0.001
Inflammatory Markers				
CRP, mg/L	7.2 (3.0–23.4)	7.0 (3.0–19.9)	8.0 (3.0–29.0)	0.508
ESR, mm/h	69.0 (33.0–99.0)	81.0 (45.8–112.0)	53.0 (23.0–83.0)	<0.001

Continuous variables are presented as median (interquartile range). Categorical variables are presented as *n* (%). *p*-values were calculated using Mann–Whitney U test for continuous variables and Chi-square test for categorical variables.

**Table 2:** Clinical presentations and final diagnoses among non-myeloma patients referred for suspected multiple myeloma (*n* = 219). Categories are not mutually exclusive; percentages are calculated over the full non-myeloma cohort (*n*/219).

Clinical Presentation/Suspected MM Mimic	*n*	%
Anemia-related conditions		
Iron deficiency anemia	39	17.8
Anemia of chronic kidney disease	42	19.2
Anemia, unspecified/multifactorial	117	53.4
Anemia, undetermined etiology (cause not identified)	6	2.7
Thalassemia trait/other hemoglobinopathy	6	2.7
Vitamin B12 deficiency	1	0.5
Autoimmune hemolytic anemia	4	1.8
Aplastic anemia	1	0.5
Renal dysfunction/chronic kidney disease		
Chronic kidney disease/renal impairment	48	21.9
Hypercalcemia-related conditions		
Hypercalcemia (any cause)	1	0.5
Bone pain/skeletal abnormalities		
Osteoporosis/degenerative bone disease	8	3.7
Elevated inflammatory markers/systemic inflammation		
Autoimmune/rheumatologic disease	25	11.4
Other chronic inflammatory conditions	9	4.1
Other hematological disorders		
Other benign hematological disorders	8	3.7
Hematological malignant/clonal disorders	12	5.5
Malignancy		
Solid malignancies	24	11.0

**Table 3:** Logistic Regression Analysis.

**Univariable Logistic Regression Analysis**			
**Variable**	**OR**	**95% CI**	***p*-value**
Total protein	5.92	3.62–9.69	<0.001
Calcium	2.59	1.77–3.80	<0.001
Creatinine	1.93	1.41–2.65	<0.001
Hemoglobin	1.88	1.43–2.45	<0.001
ESR	1.85	1.44–2.38	<0.001
Uric acid	1.62	1.25–2.10	<0.001
MCV	1.59	1.20–2.11	0.001
WBC	1.11	0.82–1.50	0.491
CRP	0.96	0.76–1.21	0.739
LDH	0.89	0.67–1.18	0.417
Age	0.71	0.56–0.90	0.004
Female sex	0.67	0.53–0.85	<0.001
Albumin	0.55	0.42–0.71	<0.001
Platelet	0.43	0.32–0.59	<0.001
Multivariable Logistic Regression Analysis *			
**Variable**	**OR**	**95% CI**	***p*-value**
Total protein	11.95	4.22–33.88	<0.001
Calcium	9.62	3.84–24.12	<0.001
Hemoglobin	4.34	2.36–8.01	<0.001
Albumin	0.16	0.08–0.33	<0.001
Creatinine	2.33	1.35–4.04	0.003
Platelet	0.46	0.26–0.81	0.007
Female sex	0.60	0.38–0.93	0.021

OR: Odds Ratio; CI: Confidence Interval. Variables sorted by OR. Continuous variables were standardized. * Only significant predictors (p < 0.05) shown. Full model results are available in Supplementary Table S1.

**Table 4:** Machine Learning Model Performance on Test Set (*n* = 127).

Model	AUC (95% CI)	Sens	Spec	PPV	NPV	Brier
Random Forest	0.955 (0.921–0.981)	0.803	0.970	0.961	0.842	0.093
Gradient Boosting	0.954 (0.918–0.984)	0.869	0.924	0.914	0.884	0.091
Ensemble (Stacking)	0.950 (0.905–0.980)	0.885	0.939	0.931	0.899	0.084
Ensemble (Weighted)	0.944 (0.895–0.978)	0.902	0.864	0.859	0.905	0.090
SVM	0.918 (0.856–0.966)	0.902	0.864	0.859	0.905	0.100
Neural Network	0.906 (0.837–0.961)	0.902	0.864	0.859	0.905	0.108
Logistic Regression	0.901 (0.832–0.958)	0.836	0.909	0.895	0.857	0.111
LASSO	0.901 (0.830–0.959)	0.852	0.924	0.912	0.871	0.110
Ridge	0.901 (0.830–0.957)	0.836	0.894	0.879	0.855	0.114

AUC: Area Under ROC Curve; Sens: Sensitivity; Spec: Specificity; PPV: Positive Predictive Value; NPV: Negative Predictive Value. Metrics at optimal threshold (Youden’s index). Models sorted by AUC. AUC 95% confidence intervals from 1000 bootstrap resamples.

**Table 5:** Simple Rule Benchmark AUCs and Paired Comparisons.

Comparison	ΔAUC	95% CI	Significant
Stacking (0.950) vs. Globulin Gap (0.769)	0.194	0.111–0.281	Yes
Stacking (0.950) vs. Total Protein (0.789)	0.176	0.101–0.259	Yes
Stacking (0.950) vs. Any-CRAB Rule (0.321)	0.642	0.571–0.717	Yes
Stacking (0.950) vs. Logistic Regression (0.901)	0.023	0.006–0.046	Yes

**Table 6:** Sensitivity Analysis: Model Performance Without Ca, Cr, and Hgb.

Model	Full Model AUC	No-CRAB AUC	ΔAUC
Logistic Regression	0.901	0.935	+0.034
Random Forest	0.955	0.946	−0.009
Ensemble Stacking	0.950	0.946	−0.004

**Table 7:** False-Negative Audit by Globulin Gap Level (Ensemble Stacking).

Globulin Gap Group	N	FN	TP	Sensitivity	Mean GG (g/dL)
Low (≤3.5 g/dL)	20	4	16	0.800 (80.0%)	2.66
Moderate (3.5–5.0 g/dL)	13	2	11	0.846 (84.6%)	4.19
High (>5.0 g/dL)	28	0	28	1.000 (100%)	6.86

## Data Availability

De-identified individual-level data are not publicly available owing to patient confidentiality and institutional restrictions. Data may be made available from the corresponding author upon reasonable request. The analysis was performed in Python 3.10 using open-source libraries (pandas, numpy, scipy, scikit-learn, statsmodels, SHAP); the analysis code is available from the corresponding author upon request.

## Supplementary Materials

The following supporting information can be downloaded at: https://www.mdpi.com/article/10.3390/diagnostics16182928/s1, Table S1: Full multivariable logistic regression results; Table S2: 10-fold cross-validation performance; Table S3: Model performance at different sensitivity thresholds; Table S4: Clinical risk stratification based on Random Forest predictions; Table S5: Hyperparameters; Table S6: Coexisting comorbidities in the non-myeloma cohort; Figure S1: Variable importance rankings for Random Forest, Gradient Boosting, and SHAP analysis; Figure S2: SHAP summary plot for the Gradient Boosting model.

## References

[1] Rajkumar S.V. (2024). Multiple myeloma: 2024 update on diagnosis, risk-stratification, and management. Am. J. Hematol..

[2] Malard F., Neri P., Bahlis N.J., Terpos E., Moukalled N., Hungria V.T.M., Manier S., Mohty M. (2024). Multiple myeloma. Nat. Rev. Dis. Primers.

[3] Rajkumar S.V. (2022). Multiple myeloma: 2022 update on diagnosis, risk stratification, and management. Am. J. Hematol..

[4] Tothong W., Tantiworawit A., Norasetthada L., Chai-Adisaksopha C., Punnachet T., Hantrakun N., Piriyakhuntorn P., Rattanathammethee T., Hantrakool S., Rattarittamrong E. (2024). Prevalence, Outcomes and Impact of Disease-Related Complications in the Survival of Multiple Myeloma Patients. Hematol. Rep..

[5] Huang J., Chan S.C., Lok V., Zhang L., Lucero-Prisno D.E., Xu W., Zheng Z.-J., Elcarte E., Withers M., Wong M.C.S. (2022). The epidemiological landscape of multiple myeloma: A global cancer registry estimate of disease burden, risk factors, and temporal trends. Lancet Haematol..

[6] Siegel R.L., Miller K.D., Fuchs H.E., Jemal A. (2022). Cancer statistics, 2022. CA Cancer J. Clin..

[7] Mikhael J., Bhutani M., Cole C.E. (2023). Multiple Myeloma for the Primary Care Provider: A Practical Review to Promote Earlier Diagnosis Among Diverse Populations. Am. J. Med..

[8] Hossain M.I., Hampson P., Nowell C., Khan S., Sen R., Sundararaman S., Adiyodi J., Basu S. (2021). An in depth analysis of factors contributing to diagnostic delay in myeloma: A retrospective UK study of patients journey from primary care to specialist secondary care. Blood.

[9] Zorlu T., Kayer M.A., Okumus N., Ulaş T., Dal M.S., Altuntas F. (2025). Challenges, Difficulties, and Delayed Diagnosis of Multiple Myeloma. Diagnostics.

[10] Silberstein J., Tuchman S., Grant S.J. (2022). What is multiple myeloma?. JAMA.

[11] Albagoush S., Shumway C., Azevedo A. (2024). Multiple Myeloma.

[12] Zamagni E. (2021). The Pathophysiology of Myeloma Bone. Management of Bone Disease and Kidney Failure in Multiple Myeloma: A Pocket Guide.

[13] Nau K.C., Lewis W.D. (2008). Multiple myeloma: Diagnosis and treatment. Am. Fam. Physician.

[14] Kyle R.A., Gertz M.A., Witzig T.E., Lust J.A., Lacy M.Q., Dispenzieri A., Fonseca R., Rajkumar S.V., Offord J.R., Larson D.R. (2003). Review of 1027 patients with newly diagnosed multiple myeloma. Mayo Clin. Proc..

[15] Cowan A.J., Green D.J., Kwok M., Lee S., Coffey D.G., Holmberg L.A., Tuazon S., Gopal A.K., Libby E.N. (2022). Diagnosis and management of multiple myeloma: A review. JAMA.

[16] Bergstrom D.J., Kotb R., Louzada M.L., Sutherland H.J., Tavoularis S., Venner C.P., Côté J., LeBlanc R., Reiman A., Sebag M. (2020). Consensus Guidelines on the Diagnosis of Multiple Myeloma and Related Disorders: Recommendations of the Myeloma Canada Research Network Consensus Guideline Consortium. Clin. Lymphoma Myeloma Leuk..

[17] Subhan A., Manoharan G. (2025). Advancing cancer care through artificial intelligence: From innovative models to clinical decision-making and regulatory integration. Clin. Cancer Bull..

[18] Lipkova J., Chen R.J., Chen B., Lu M.Y., Barbieri M., Shao D., Vaidya A.J., Chen C., Zhuang L., Williamson D.F. (2022). Artificial intelligence for multimodal data integration in oncology. Cancer Cell.

[19] Santos C.S., Amorim-Lopes M. (2025). Externally validated and clinically useful machine learning algorithms to support patient-related decision-making in oncology: A scoping review. BMC Med. Res. Methodol..

[20] Alhamrani S.Q., Ball G.R., El-Sherif A.A., Ahmed S., Mousa N.O., Alghorayed S.A., Alatawi N.A., Ali A.M., Alqahtani F.A., Gabre R.M. (2025). Machine Learning for Multi-Omics Characterization of Blood Cancers: A Systematic Review. Cells.

[21] Amjad H., Hussain Z., Hasan M., Ul Hassan M. (2025). Machine learning-based models for screening of anemia and leukemia using features of complete blood count reports. Sci. Rep..

[22] Yu C., Peng Y.Y., Liu L., Wang X., Xiao Q. (2022). Leukemia can be Effectively Early Predicted in Routine Physical Examination with the Assistance of Machine Learning Models. J. Heal. Eng..

[23] Cheng Z.J., Li H., Liu M., Fu X., Liu L., Liang Z., Gan H., Sun B. (2024). Artificial intelligence reveals the predictions of hematological indexes in children with acute leukemia. BMC Cancer.

[24] Liu J., Gou Y., Yang W., Wang H., Zhang J., Wu S., Liu S., Tao T., Tang Y., Yang C. (2025). Development and application of machine learning models for hematological disease diagnosis using routine laboratory parameters: A user-friendly diagnostic platform. Front. Med..

[25] Sutanto H., Romadhon P.Z., Fatmawati V.R., Waitupu A., Ansharullah B.A., Rachma B., Elisa E., Pratiwi L., Adytia G.J. (2025). Multiple Myeloma and Precursor Plasma Cell Disorders: From Emerging Driver Mutations to Current and Future Therapeutic Strategies. Hemato.

[26] Cai Y., Zhao Y., Dai Q., Xu M., Xu X., Xia W. (2021). Prognostic value of the albumin-globulin ratio and albumin-globulin score in patients with multiple myeloma. J. Int. Med. Res..

[27] Lv X.M., Yan L.W., Yu G.Q., Chen Z.Z., Xiao L., Yu H.L. (2025). The Diagnostic and Differential Value of the Serum Albumin-to-Globulin Ratio in Multiple Myeloma. Clin. Lab..

[28] Vakiti A., Anastasopoulou C., Mewawalla P. (2023). Malignancy-Related Hypercalcemia.

[29] Walker M.D., Shane E. (2022). Hypercalcemia: A review. JAMA.

[30] Calabrese V., Messina R.M., Cernaro V., Farina A., Di Pietro Y., Gembillo G., Longhitano E., Casuscelli C., Taverna G., Santoro D. (2025). Hypercalcemia: A Practice Overview of Its Diagnosis and Causes. Kidney Dial..

[31] Marino S., Roodman G.D. (2017). Multiple Myeloma and Bone: The Fatal Interaction. Cold Spring Harb. Perspect. Med..

[32] Harmer D., Falank C., Reagan M.R. (2019). Interleukin-6 Interweaves the Bone Marrow Microenvironment, Bone Loss, and Multiple Myeloma. Front. Endocrinol..

[33] van de Donk N.W.C.J., Pawlyn C., Yong K.L. (2021). Multiple myeloma. Lancet.

[34] Nairz M., Theurl I., Wolf D., Weiss G. (2016). Iron deficiency or anemia of inflammation?: Differential diagnosis and mechanisms of anemia of inflammation. Wien. Med. Wochenschr..

[35] Shanbhag S., Naik R.P., Busby-Whitehead J., Durso S.C., Arenson C., Elon R., Palmer M.H., Reichel W. (2022). Anemia and Other Hematological Problems. Reichel’s Care of the Elderly: Clinical Aspects of Aging.

[36] Hashmi M.F., Shaikh H., Rout P. (2024). Anemia of Chronic Kidney Disease.

[37] Evstatiev R. (2016). Iron deficiency, thrombocytosis and thromboembolism. Wien. Med. Wochenschr..

[38] Rokkam V.R., Killeen R.B., Kotagiri R. (2024). Secondary Thrombocytosis.

[39] Charalampous C., Goel U., Kapoor P., Binder M., Buadi F., Dingli D., Dispenzieri A., Fonder A., Gertz M., Gonsalves W. (2024). Association of Thrombocytopenia With Disease Burden, High-Risk Cytogenetics, and Survival in Newly Diagnosed Multiple Myeloma Patients Treated With Novel Therapies. Clin. Lymphoma Myeloma Leuk..

[40] Pires A.M., Barreto J.P., Caetano J., Soares M.J., Geraldes C., Fernandes B., Coucelo M., Chacim S., Coelho H., Correia C. (2025). Multiple Myeloma Laboratory Diagnostics Made Simple: Practical Insights and Key Recommendations. J. Clin. Med..

[41] Ferri G.M., Yildirim C., Do N.V., Brophy M.T., Munshi N.C., Edwards C.V., Fillmore N.R. (2025). Moderate-Severe Thrombocytopenia Portends Poor Outcomes in Multiple Myeloma. eJHaem.

[42] Naeem A., Amar S., Mehta D., Malik M.N. (2019). Thrombocytosis as an Initial Presentation of Plasma Cell Neoplasm: A Case Report. Cureus.

[43] Yao B., Liu Y., Wu Y., Mao S., Zhang H., Jiang L., Fei C., Wang S., Tong J., Wu J. (2024). Common laboratory results-based artificial intelligence analysis achieves accurate classification of plasma cell dyscrasias. PeerJ.

[44] Li M., Jia S., Wu H., Shou C., Song X., Peng Y., Wang H., Wang Y., Tong X., Chen Y. (2025). A differential diagnostic model based on immunological evaluation and routine laboratory tests: Distinguishing multiple myeloma from other disorders with aberrant immunoglobulin elevation. Discov. Oncol..

[45] Breiman L. (2001). Random Forests. Mach. Learn..

[46] Friedman J.H. (2001). Greedy function approximation: A gradient boosting machine. Ann. Stat..

[47] Lundberg S.M., Lee S.-I. (2017). A unified approach to interpreting model predictions. Proceedings of the Advances in Neural Information Processing Systems.

[48] Lundberg S.M., Erion G., Chen H., DeGrave A., Prutkin J.M., Nair B., Katz R., Himmelfarb J., Bansal N., Lee S.-I. (2020). From Local Explanations to Global Understanding with Explainable AI for Trees. Nat. Mach. Intell..

[49] Friese C.R., Abel G.A., Magazu L.S., Neville B.A., Richardson L.C., Earle C.C. (2009). Diagnostic delay and complications for older adults with multiple myeloma. Leuk. Lymphoma.

[50] Koshiaris C., Oke J., Abel L., Nicholson B.D., Ramasamy K., Bruel A.V.D. (2018). Quantifying intervals to diagnosis in myeloma: A systematic review and meta-analysis. BMJ Open.

[51] McCurdy A., Mian H., Rosenberg H. (2022). Just the facts: How to diagnose and manage patients with multiple myeloma in the emergency department?. Can. J. Emerg. Med..

