# Supplementary material for: Routine Laboratory-Based Machine Learning for Discriminating Multiple Myeloma from Clinical Mimickers: Development and Internal Validation of a Diagnostic Prediction Model
Source: Diagnostics (Basel). 2026 Sep 10;16(18):2928. doi: 10.3390/diagnostics16182928 (PMC13605497; doi:10.3390/diagnostics16182928)
Supplement: Supplementary file 1 [file diagnostics-16-02928-s001.zip › diagnostics-4525471-supplementary.pdf]

Supplementary Materials

Supplementary Table S1. Full Multivariable Logistic Regression Results

Variable	Coefficient	OR	95% CI	p-value
Total protein	2.481	11.95	4.22-33.88	<0.001
Calcium	2.264	9.62	3.84-24.12	<0.001
Hemoglobin	1.468	4.34	2.36-8.01	<0.001
Albumin	-1.852	0.16	0.08-0.33	<0.001
Creatinine	0.847	2.33	1.35-4.04	0.003
Platelet	-0.779	0.46	0.26-0.81	0.007
Female sex	-0.519	0.60	0.38-0.93	0.021
Age	-0.434	0.65	0.40-1.06	0.083
Uric acid	0.397	1.49	0.81-2.72	0.198
WBC	-0.380	0.68	0.29-1.60	0.382
MCV	0.183	1.20	0.77-1.88	0.422
ESR	-0.120	0.89	0.49-1.60	0.691
LDH	0.092	1.10	0.57-2.12	0.785
CRP	-0.017	0.98	0.61-1.59	0.946

Supplementary Table S2. 10-Fold Cross-Validation Performance

Model	10-fold CV AUC	SD	Repeated 10×10 CV AUC	SD	Brier
Random Forest	0.949	0.033	0.950	0.032	0.097
SVM	0.945	0.032	0.948	0.036	0.088
Logistic Regression	0.938	0.046	0.942	0.039	0.095
Ridge	0.937	0.049	0.941	0.037	0.105
Gradient Boosting	0.928	0.039	0.936	0.038	0.104
LASSO	0.924	0.052	0.940	0.041	0.115
Neural Network	0.872	0.073	0.877	0.071	0.162

AUC: Area Under ROC Curve; SD: Standard Deviation; Brier: Mean Brier Score. Repeated 10×10 CV: 10-fold cross-validation repeated 10 times (100 total iterations). Models sorted by 10-fold CV AUC.

Supplementary Table S3. Model Performance at Different Sensitivity Thresholds

Model	Threshold	Sens	Spec	PPV	NPV	Accuracy
Optimal Threshold (Youden's Index)						
Random Forest	0.501	0.869	0.909	0.898	0.882	0.890
Gradient Boosting	0.561	0.885	0.924	0.915	0.897	0.906
Ensemble (Stacking)	0.589	0.885	0.939	0.931	0.899	0.913
≥90% Sensitivity Threshold						
Random Forest	0.367	0.918	0.818	0.824	0.915	0.866
Ensemble (Stacking)	0.410	0.902	0.879	0.873	0.906	0.890
≥95% Sensitivity Threshold (Screening)						
Random Forest	0.339	0.951	0.773	0.795	0.944	0.858
Gradient Boosting	0.126	0.951	0.788	0.806	0.945	0.866
Ensemble (Stacking)	0.191	0.951	0.727	0.763	0.941	0.835

Sens: Sensitivity; Spec: Specificity; PPV: Positive Predictive Value; NPV: Negative Predictive Value. High-sensitivity thresholds recommended for screening to minimize missed diagnoses.

Supplementary Table S4. Clinical Risk Stratification Based on Random Forest Predictions

Risk Category	N	Myeloma N	Myeloma Rate (%)	95% CI (%)
Very Low (<20%)	39	1	2.6	0.4-12.6
Low (20-40%)	21	4	19	15.6-50.9
Moderate (40-60%)	14	7	50	16.8-68.7
High (60-80%)	13	10	76.9	59.0-93.8
Very High (>80%)	40	39	97.5	85.8-99.5

CI: Wilson score 95% confidence interval. Risk categories based on predicted probability from Random Forest model on test set.

Supplementary Table S5. Hyperparameters

Model	Parameter	Final Value
LASSO Logistic Regression	Regularization parameter (C)	0.2336
	Selected variables	Sex, RBC, hemoglobin, platelet count, MCV, creatinine, total protein, albumin, calcium, uric acid, ESR
Ridge Logistic Regression	Regularization parameter (C)	0.2336

Random Forest	Number of trees (n_estimators)	100
	Maximum tree depth (max_depth)	10
	Minimum samples per split	5
	Minimum samples per leaf	2
Gradient Boosting	Number of estimators	150
	Learning rate	0.10
	Maximum depth	5
	Subsample ratio	0.80
Support Vector Machine	Kernel	Radial basis function (RBF)
	Regularization parameter (C)	10
	Kernel coefficient (gamma)	0.01
Neural Network	Hidden layer size	(100,)
	Regularization parameter (alpha)	0.01

Supplementary Figures

Supplementary Figure S1. Variable importance rankings for Random Forest, Gradient Boosting, and SHAP analysis.

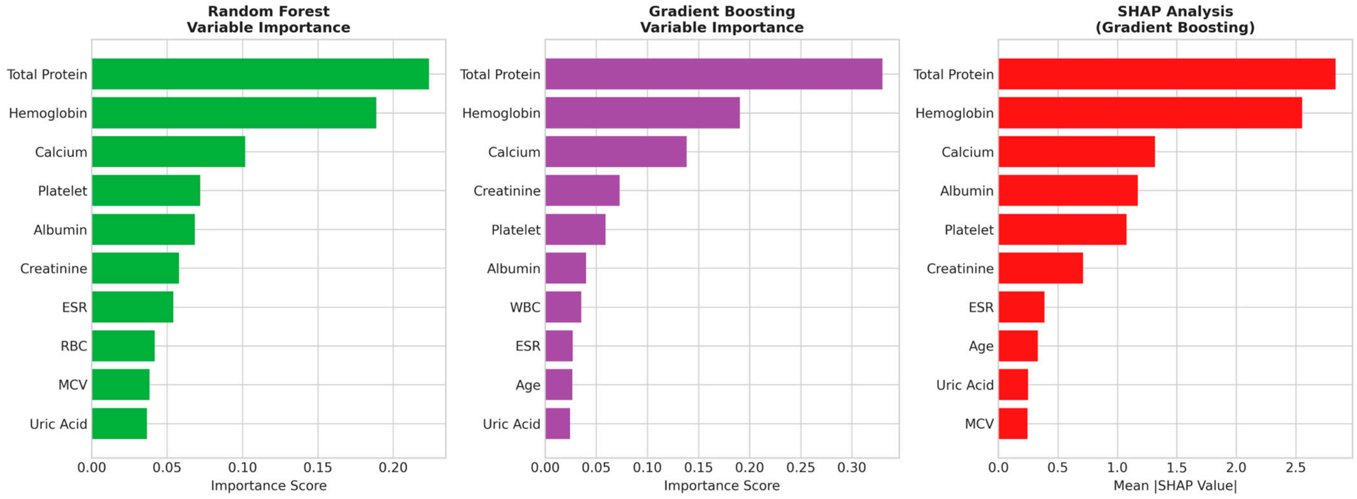

Supplementary Figure S2. SHAP summary plot for the Gradient Boosting model.

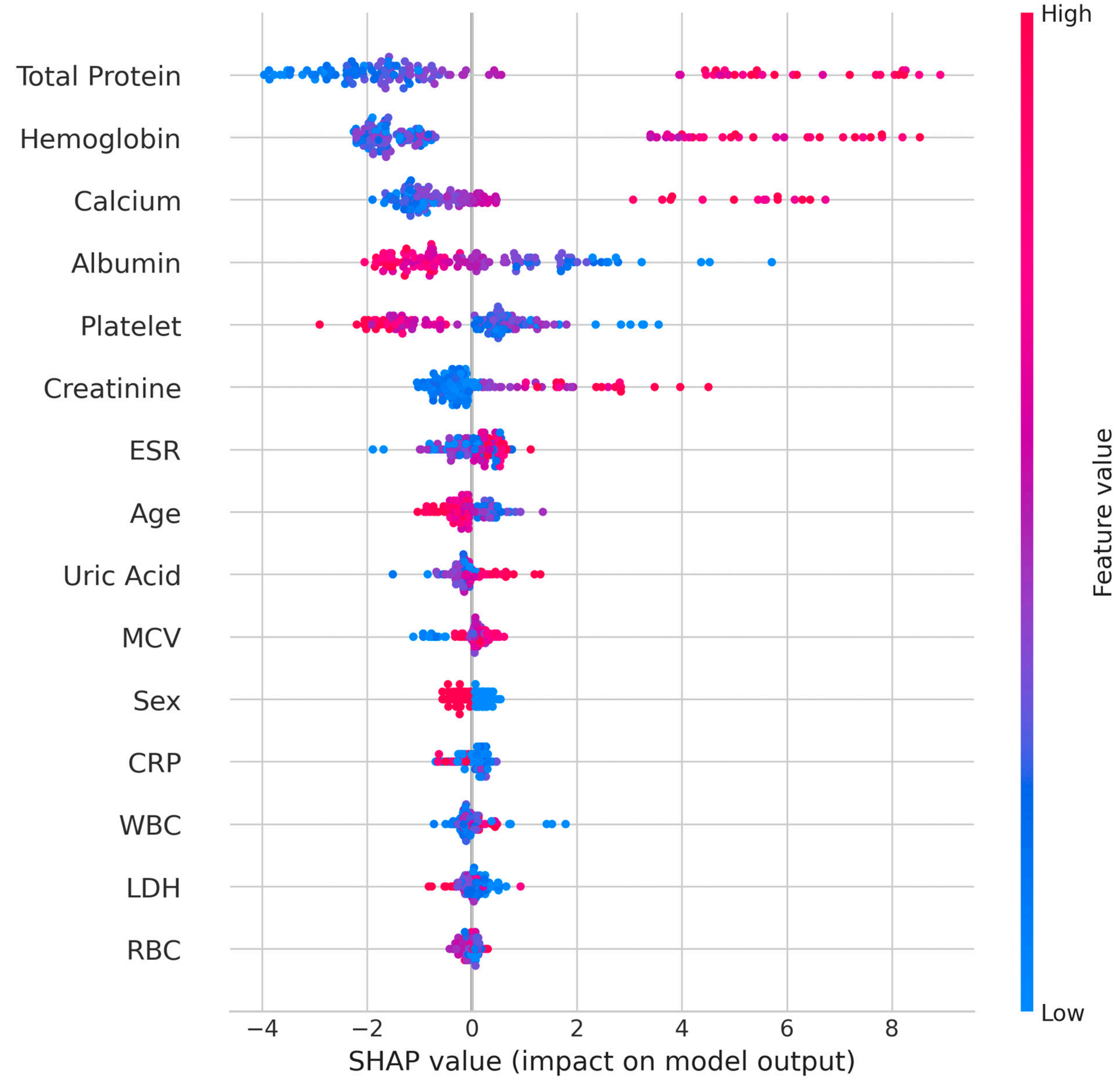

Supplementary Table S6. Coexisting comorbidities in the non-myeloma cohort (contextual; not myeloma mimics).

These conditions were recorded as background comorbidities and do not themselves mimic multiple myeloma; they are reported for completeness and to characterize the referral population. Percentages are calculated over the full non-myeloma cohort (n/219).

**Coexisting comorbidity (not an MM mimic)**	**n**	**%**
Hypertension	119	54.3
Cardiovascular disease	89	40.6
Diabetes / other metabolic-endocrine disease	89	40.6
Neurologic disease	37	16.9
Hyperlipidemia	20	9.1
Chronic respiratory disease	18	8.2

Notes on control-group composition

- Near-neighbor plasma-cell disorders were not present in the control group. No cases of MGUS, smoldering multiple myeloma, solitary plasmacytoma, or AL amyloidosis were represented; the single patient with amyloidosis had non–plasma-cell (cardiac/AA-type) disease. The non-myeloma group was therefore not enriched by excluding difficult near-neighbor diagnoses.
- Clonal/malignant hematological near-neighbors were retained as controls, including myelodysplastic syndrome (MDS), primary myelofibrosis, chronic myeloid leukemia, and B-cell lymphomas (marginal-zone and non-Hodgkin). These represent genuinely challenging discriminations rather than trivially separable controls.
- Hypercalcemia was essentially absent among non-myeloma patients (n=1), indicating that the model's discrimination is not attributable to a hypercalcemia imbalance between groups.
- Anemia was the dominant referral trigger. Where a specific etiology was documented it is listed; the remaining anemic patients are grouped as unspecified/multifactorial, and six patients referred for anemia in whom no cause was identified are listed separately.
